# Supplementary material for: The push–pull intercrop Desmodium does not repel, but intercepts and kills pests
Source: eLife. 2024 Mar 13;13:e88695. doi: 10.7554/eLife.88695 (PMC11021049; doi:10.7554/eLife.88695)
Supplement: MDAR checklist [file elife-88695-mdarchecklist1.pdf]

eLife

Westbrook Centre
Milton Road
Cambridge CB4 1YG, UK

P 01223 855340
W elifesciences.org
T @elife

Materials Design Analysis Reporting (MDAR) Checklist for Authors

The MDAR framework establishes a minimum set of requirements in transparent reporting mainly applicable to studies in the life sciences.

eLife asks authors to **provide detailed information within their article** to facilitate the interpretation and replication of their work. Authors can also upload supporting materials to comply with relevant reporting guidelines for health-related research (see EQUATOR Network), life science research (see the BioSharing Information Resource), or animal research (see the ARRIVE Guidelines and the STRANGE Framework; for details, see *eLife*'s Journal Policies). Where applicable, authors should refer to any relevant reporting standards materials in this form.

For all that apply, please note **where in the article** the information is provided. Please note that we also collect information about data availability and ethics in the submission form.

Materials:

Newly created materials	Indicate where provided: section/figure legend	N/A
The manuscript includes a dedicated "materials availability statement" providing transparent disclosure about availability of newly created materials including details on how materials can be accessed and describing any restrictions on access.		X

Antibodies	Indicate where provided: section/figure legend	N/A
For commercial reagents, provide supplier name, catalogue number and RRID, if available.		X

DNA and RNA sequences	Indicate where provided: section/figure legend	N/A
Short novel DNA or RNA including primers, probes: Sequences should be included or deposited in a public repository.		X

Cell materials	Indicate where provided: section/figure legend	N/A
Cell lines: Provide species information, strain. Provide accession number in repository OR supplier name, catalog number, clone number, OR RRID.		X
Primary cultures: Provide species, strain, sex of origin, genetic modification status.		X

Experimental animals	Indicate where provided: section/figure legend	N/A
Laboratory animals or Model organisms: Provide species, strain, sex, age, genetic modification status. Provide accession number in repository OR supplier name, catalog number, clone number, OR RRID.	Described in section Materials and methods under subsection Insect rearing	
Animal observed in or captured from the field: Provide species, sex, and age where possible.		X

Plants and microbes	Indicate where provided: section/figure legend	N/A
Plants: provide species and strain, ecotype and cultivar where relevant, unique accession number if available, and source (including location for collected wild specimens).	Described in section Materials and methods under subsection Plants	
Microbes: provide species and strain, unique accession number if available, and source.	Described in section Materials and methods under subsection Plants<br><br>Only commercially avaialable microbial inoculates were used, which were the following:<br><br>Rhizobia inoculant for Phaseolus, and soy beans from Samenfest GmbH Freiburg, Germany: Rhizobium leguminosarum, Bradyrhizobium japonicum)<br><br>Mycorrhizal inoculant:<br>Wildroot Organic Inc., Texas: mixture of Glomus intraradices, G. etunicatum, G. monosporum, G. deserticola, G. clarum, Paraglomus brasilianum, Gigaspora margarita, Rhizopogon villosulus, R. lutcolus, R. amylopogon, R. fulvigleba, Pisolithus tinctorius, Scleroderma cepa and S. citrinum.	

Human research participants	Indicate where provided: section/figure legend) or state if these demographics were not collected	N/A
If collected and within the bounds of privacy constraints report on age, sex, gender and ethnicity for all study participants.		X

Design:

Study protocol	Indicate where provided: section/figure legend	N/A

If the study protocol has been pre-registered, provide DOI. For clinical trials, provide the trial registration number OR cite DOI.		X

Laboratory protocol	Indicate where provided: section/figure legend	N/A
Provide DOI OR other citation details if detailed step-by-step protocols are available.		X

Experimental study design (statistics details) *		
**For in vivo studies: State whether and how the following have been done**	**Indicate where provided: section/figure legend. If it could have been done, but was not, write “not done”**	**N/A**
Sample size determination	The sample sizes used in this experiment were in line with sample sizes used in earlier studies.<br>Since the aim of this study was to compare volatile emissions of plants belonging to different species the goal was to maximise power rather than to detect at specific effect sizes.	
Randomisation	The plant positions in the greenhouse were randomised weekly during the plant growth period and plants were sampled in parallel. The plant-choice positions were randomized in the behavioral bioassays.	
Blinding	Blinding was not possible in the volatile collection experiments and behavioral experiments.	
Inclusion/exclusion criteria	Four samples were exluded from wind tunnel bioassay analysis, and 4 samples from cage bioassays, when females did not lay any eggs during the entire period.	

Sample definition and in-laboratory replication	Indicate where provided: section/figure legend	N/A
State number of times the experiment was replicated in the laboratory.	Replication numbers are stated in the description of each experiment in the Materials and methods section and figure legend.	
Define whether data describe technical or biological replicates.	The replicates described are biological replicates as plants and insects were not reused for experiments.	

Ethics	Indicate where provided: section/submission form	N/A
Studies involving human participants: State details of authority granting ethics approval (IRB or equivalent committee(s), provide reference number for approval.		X
Studies involving experimental animals: State details of authority granting ethics approval (IRB or equivalent committee(s), provide reference number for approval.		X
Studies involving specimen and field samples: State if relevant permits obtained, provide details of authority approving study; if none were required, explain why.		X

Dual Use Research of Concern (DURC)	Indicate where provided: section/submission form	N/A
If study is subject to dual use research of concern regulations, state the authority granting approval and reference number for the regulatory approval.		X

Analysis:

Attrition	Indicate where provided: section/figure legend	N/A
Describe whether exclusion criteria were pre-established. Report if sample or data points were omitted from analysis. If yes, report if this was due to attrition or intentional exclusion and provide justification.	Four samples were exluded from wind tunnel bioassay analysis, and 4 samples from cage bioassays, when females did not lay any eggs during the entire period of the experiment.	

Statistics	Indicate where provided: section/figure legend	N/A
Describe statistical tests used and justify choice of tests.		X

Data availability	Indicate where provided: section/submission form	N/A
For newly created and reused datasets, the manuscript includes a data availability statement that provides details for access (or notes restrictions on access).		X

When newly created datasets are publicly available, provide accession number in repository OR DOI and licensing details where available.	The dataset accessibility is included in the manuscript, data is available on figshare: https://figshare.com/articles/dataset/The_push-pull_intercrop_Desmodium_does_not_repel_but_intercepts_and_kills_pest/19297730	
If reused data is publicly available provide accession number in repository OR DOI, OR URL, OR citation.		X

Code availability	Indicate where provided: section/figure legend	N/A
For any computer code/software/mathematical algorithms essential for replicating the main findings of the study, whether newly generated or re-used, the manuscript includes a data availability statement that provides details for access or notes restrictions.	he identification and verification of volatile components was done using Agilent Mass Hunter B.08.00 and NIST MS Search 2.4 using Wiley 12 and NIST11 MS libraries.<br>The statistical analysis was performed in R v3.6.1 including packages:<br>vegan v2.5-7,<br>lme4 v1.1-27.1,<br>DHARMa v0.4.4,<br>survival v.3.2-13,<br>surviver v.0.4.9.	
Where newly generated code is publicly available, provide accession number in repository, OR DOI OR URL and licensing details where available. State any restrictions on code availability or accessibility.		X
If reused code is publicly available provide accession number in repository OR DOI OR URL, OR citation.		X

Reporting:

The MDAR framework recommends adoption of discipline-specific guidelines, established and endorsed through community initiatives.

Adherence to community standards	Indicate where provided: section/figure legend	N/A
State if relevant guidelines (e.g., ICMJE, MIBBI, ARRIVE, STRANGE) have been followed, and whether a checklist (e.g., CONSORT, PRISMA, ARRIVE) is provided with the manuscript.		X
